# Insulin receptor membrane retention by a traceable chimeric mutant

**DOI:** 10.1186/1478-811X-11-45

**Published:** 2013-06-27

**Authors:** Jimena Giudice, Elizabeth A Jares-Erijman, Federico Coluccio Leskow

**Affiliations:** 1 Departamento de Química Biológica, Facultad de Ciencias Exactas y Naturales (FCEN), Universidad de Buenos Aires (UBA) IQUIBICEN, CONICET, Intendente Güiraldes 2160, Ciudad Universitaria, C1428EGA Buenos Aires, Argentina; 2 Departamento de Química Orgánica, FCEN UBA CIHIDECAR, CONICET,C1428EGA Buenos Aires, Argentina; 3 Departamento de Ciencias Básicas, Universidad Nacional de Luján, Buenos Aires, Argentina; 4 Present address: Department of Pathology and Immunology, Baylor College of Medicine, One Baylor Plaza, Houston, TX 77030 USA

**Keywords:** Insulin receptor, Membrane retention, Dominant negative, Endocytosis

## Abstract

**Background:**

The insulin receptor (IR) regulates glucose homeostasis, cell growth and differentiation. It has been hypothesized that the specific signaling characteristics of IR are in part determined by ligand-receptor complexes localization. Downstream signaling could be triggered from the plasma membrane or from endosomes. Regulation of activated receptor's internalization has been proposed as the mechanism responsible for the differential isoform and ligand-specific signaling.

**Results:**

We generated a traceable IR chimera that allows the labeling of the receptor at the cell surface. This mutant binds insulin but fails to get activated and internalized. However, the mutant heterodimerizes with wild type IR inhibiting its auto-phosphorylation and blocking its internalization. IR membrane retention attenuates AP-1 transcriptional activation favoring Akt activation.

**Conclusions:**

These results suggest that the mutant acts as a selective dominant negative blocking IR internalization-mediated signaling.

## Background

Insulin receptor (IR) is a tetrameric tyrosine kinase receptor involved on glucose homeostasis, cell growth and differentiation. Two IR variants are produced in mammals by alternative splicing: IR-A lacking exon 11 and the full length IR-B [1,2]. While IR-B is widely expressed in adult tissues, embryos predominantly express IR-A where it functions as a regulator of cellular proliferation and differentiation [3]. Alterations in the ratio of IR isoform expression have been associated with cellular dysregulation and disease. Some reports showed that in diabetic patients there are differences at the mRNA level in the IR-A/IR-B ratio in skeletal muscle [4,5], however this was not observed by others [6-8]. Cancer cells commonly express the IR-A subtype [9-12]. In addition, there are differences in the activation and signaling events between the two isoforms, indicating specific functions [13-15]. Using an elegant harmonic oscillator mathematical model, Knudson et al. reported that insulin has 1.5-fold higher affinity and a 2-fold higher dissociation rate for IR-A, than for IR-B [16]. On the other hand, IR-B binds insulin with higher affinity than for insulin like growth factor II (IGF-II) [3]. Additionally, it has been recently shown that IR-A binds IGF-II with a lower affinity than insulin [17], in contrast with a previous report informing similar affinities [3]. Over-expression of IR-A was suggested to contribute to the modulation of insulin and IGF responses in different tissues and during cancer progression [18]. Hybrid receptors are formed in cells where IR and IGF-I receptor (IGF-IR) are co-expressed and this is common in tumor tissues. Thus, the relative expression levels of IR-A, IR-B and IGF-IR affect sensitivity to ligands (IGF-I, IGF-II, insulin).

The link between metabolic and mitogenic effects of insulin are clinically relevant since, for instance, insulin treated type 2 diabetics are more likely to develop tumors [19]. Furthermore, their cancer risk may be modified by different treatments [20,21] and modified insulin analogues with distinct receptor-binding characteristics showed different mitogenic potencies in cell lines and animals [22,23]. Increase in mitogenicity was observed in analogues with lower dissociation constant from the IR [24].

Binding of insulin to the IR leads to its kinase activation, promoting the phosphorylation in *cis* and *trans* of tyrosine residues. Phosphorylated IR activates downstream cascades affecting glucose uptake, metabolism, cell growth, differentiation, gene expression and cell cycle progression. It has been postulated that the balance between these effects is affected by the receptor localization and redistribution. Activated ligand-receptor complexes are internalized into endosomes where the IR kinase would be able to phosphorylate substrates that are spatially distinct from those accessible at the plasma membrane affecting the balance between metabolic and mitogenic response. At the cell membrane activated IR recruits IRS-1 and Akt leading to the translocation of the glucose transporter and the activation of the metabolic response [25]. On the other hand, endosomes have long been proposed as signaling platforms [26], and activated IR internalization is required for the activation of the Shc/MAPK leading to the activation of early response genes and the activation of the activating protein transcription factors (AP-1), a hallmark of the mitogenic response [27-29].

Here we describe an IR-B chimera that can be modified exclusively at the plasma membrane by inserting three copies in tandem of the A1 tag [30] in the second Fibronectin type III domain (FnIII-2) of IR-B. This chimera binds insulin but fails to be activated or internalized. We show that it acts as a selective dominant negative IR by retaining the activated receptor at the plasma membrane, blocking AP-1 induction but maintaining Akt activation.

## Results and discussion

Recently we studied insulin and IGF-II endocytosis dynamics in living cells through IR-B [31,32]. Here we report novel IR-B chimeras containing an extracellular tag suitable to be covalently modified at the plasma membrane. The tag, cloned into the IR-B sequence, is specifically recognized by the acyl carrier protein (ACP) syntase (ACP-S) which transfers a 4’-phosphopantetheine group from the Coenzyme A (CoA) to a conserved serine inside the A1 sequence. This approach allowed us to label IR-B with small fluorescent dyes or biotin exclusively at the plasma membrane and the modification showed no effect on insulin binding. These chimeras bind insulin but fail to be activated being retained at the cell surface. Co-expression with wild type IR showed that these mutants function as selective dominant negatives inhibiting the induction of AP-1 activity by insulin without affecting Akt activation.

### Imaging of IR exclusively at the plasma membrane

**Figure 1:**
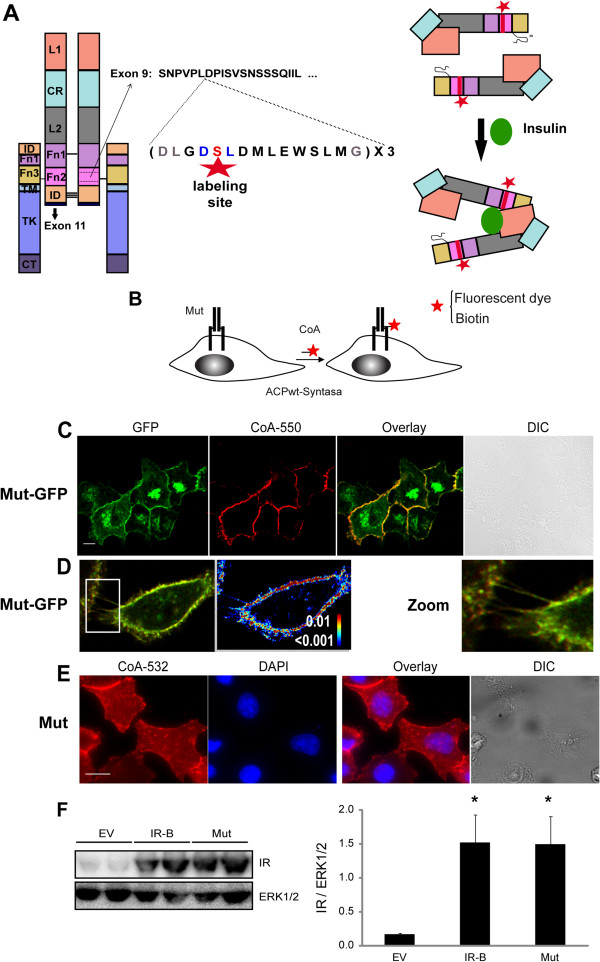
**Covalent modification of IR-B at the plasma membrane. A**. Tag position and insulin binding scheme according to proposed model [33,34]. L1, Large leucine rich domain 1; CR, cysteine rich domain; L2, large leucine rich domain 2; FnIII domains 1, 2 and 3; ID, inter-domain; TM, transmembrane domain; TK, tyrosine kinase; CT, C-terminus. Inserted tag shows: in grey, residues added for cloning reasons; in red, Ser recognized by ACP-S; in blue, conserved flanking amino acids for enzyme recognition [30]. **B**. Labeling strategy. **C**-**E**. HeLa cells expressing Mut-GFP (**C**-**D**) or Mut (**E**) were labeled with 0.2 μM ACP-S and 1 μM fluorescent CoA and imaged by confocal (Mut-GFP) or wide field microscopy (Mut). Scale bars: 10 μm. **D**. Manders map. **F**. Cells transiently expressing wild type IR-B or the mutant were assayed by Western blot 48 h after transfection using anti-IR-β-subunit or anti-ERK1/2. Quantification was performed by densitometry. Results are expressed as the mean ± s.e.m. (*: *p*≤0.05; *n*≥3).

We generated the plasmids pcDNA3-IR-B-A1×3 (Mut) and pcDNA3-IR-B-A1×3-GFP (Mut-GFP) by fusing the A1 tag (GDSLDMLEWSLM) [30] three times in tandem into the IR-B at the position 626 of the amino acids sequence (exon 9). This position is localized on the FnIII-2 domain of IR-B (Figure 1A), and does not contain known residues involved in pathological mutations, glycosilations sites, or cysteines which are important in post-transductional modifications. We hypothesize that this position does not affect insulin binding since it is located inside a domain that is not involved in the ligand-receptor ligand contact [33,34]. Other chimeras tagged on the first large Leucine rich domain (L1) showed correct expression but failed to bind insulin (unpublished data). The new chimeras allowed us to label the IR extracellular portion in living cells following the protocol showed in Figure 1B. Cells expressing the tagged IR mutants were labeled using ACP-S which transfers a 4’-phosphopantetheine group from the CoA to the A1 sequence (in red, Figure 1A). When the membrane impermeable CoA is covalently bound to a fluorescent or a biotinylated group by the sulfhydryl extreme this modification is transferred to the tagged protein exposed to the extracellular medium.

Living HeLa cells expressing the chimeras (Figure 1C-E) were labeled with 0.2 μM ACP-S and 1 μM CoA conjugated with the fluorescent ATTO-532 (CoA-532) or CoA-550. Both mutants localize correctly at the plasma membrane. Co-localization between green fluorescent protein (GFP) and CoA-550 signals was evaluated by Manders analysis: CoA-550 associated pixels were localized to the plasma membrane and co-localized with GFP signal (Figure 1E). Western blot experiments showed the correct molecular weight and similar levels of expression than wild type IR-B (Figure 1F). It should be noted that expression levels of endogenous IR in HeLa cells are bellow the detection threshold of our experimental approach as we have previously reported [32].

### Tagged IR-B binds insulin but fails to be activated

**Figure 2:**
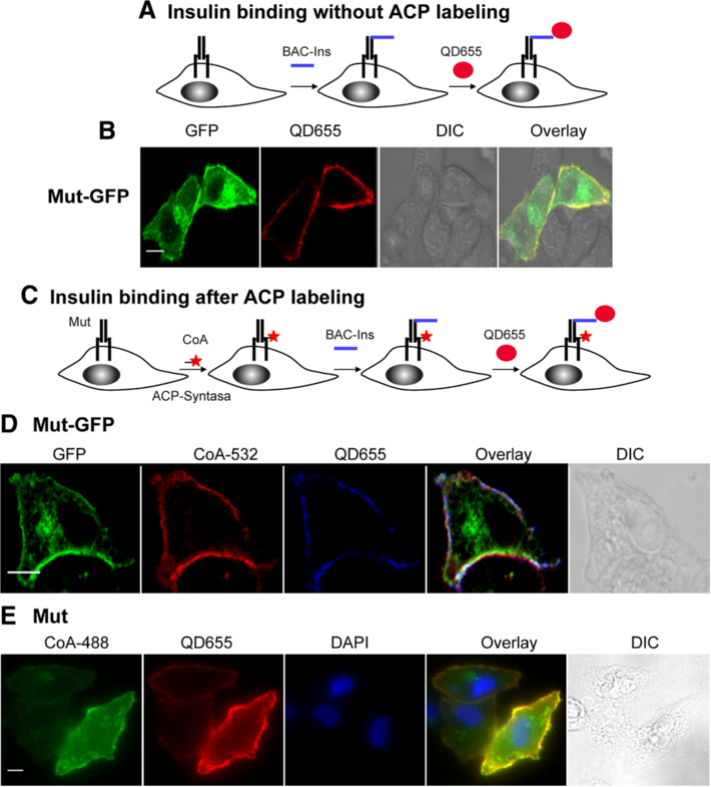
**Insulin binding assays. A**. Strategy for insulin binding and QD655 labeling. **B**. HeLa cells expressing Mut-GFP were incubated with 50 nM BAC-Ins and 1 nM QD655, fixed and imaged by confocal microscopy. **C**. Insulin binging after ACP labeling. **D**-**E**. Cells expressing the mutants were labeled with 0.2 μM ACP-S and 1 μM fluorescent CoA and then with 50 nM BAC-Ins and 1 nM QD655. Imaging was performed by confocal (**D**) or wide field microscopy (**E**). Scale bars: 10 μm.

Next, we studied the ability of the tagged receptors to bind insulin. Cells were incubated with 50 nM biotin amido caproyl insulin (BAC-Ins) for 15 min and then with 1 nM streptavidin (SA) conjugated quantum dots (QD) 655 (QD655) (maximum emission pick: 655 nm) for 10 min at room temperature [30]. QD incubation was performed with or without previous ACP labeling (Figure 2). Cells expressing Mut-GFP showed insulin binding with or without ACP labeling (Figure 2A-D). Similarly, insulin binding was observed in cells expressing Mut previously labeled with CoA-488 (Figure 2E). Binding have shown to be specific since non-transfected cells did not show QD655 signal (non-transfected cells at the same observation field and data not shown).

**Figure 3:**
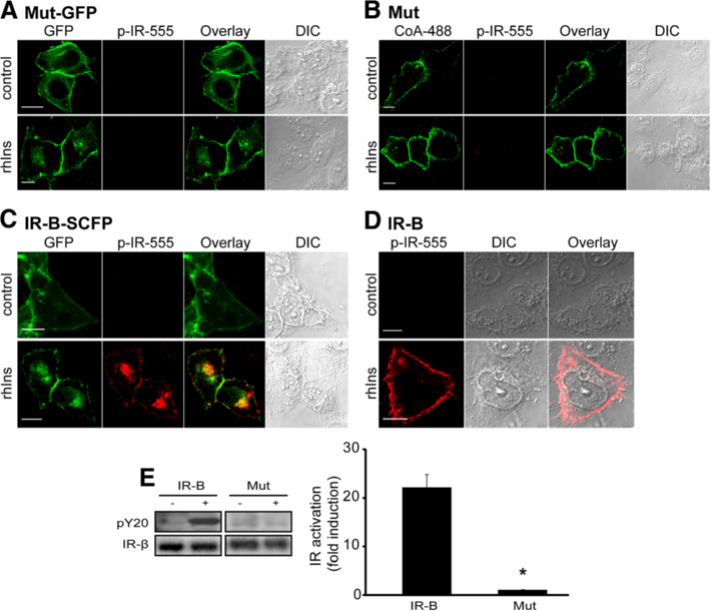
**Chimeras characterization. A**. HeLa cells expressing Mut-GFP (**A**), Mut (**B**), IR-B-SCFP (**C**) or IR-B (**D**) were stimulated with 100 nM rhIns for 10 min and fixed. Immunofluorescence assays were performed with anti-phospho-IR and a secondary antibody conjugated with Alexa fluor 555. Cells expressing Mut were labeled with 0.2 μM ACP-S and 1 μM CoA-488 before stimulation. **E**. Cells expressing wt IR-B or the mutant were stimulated with 100 nM rhIns for 5 min and lysates were analyzed by Western blot using anti-pY20 and anti-IR-β subunit. Quantification was performed by densitometry. The ratio pY20 / IR was measured for each lane and fold induction was calculated with respect to basal levels. Results are expressed as the mean ± s.e.m. (*: *p*=0.002; *n*≥3). ´p´ means phospho-antibodies.

Activation of the tagged IR in response to insulin was analyzed by immunofluorescence using a specific anti-phospho-IR-β subunit (Tyr1361) antibody and by Western blot using an anti-phospho-tyrosine (pY20) antibody. While IR-B and IR-B fused to the super cyan fluorescent protein (SCFP) (IR-B-SCFP) could be activated after 10 min by recombinant human insulin (rhIns), both mutants did not show any activation signal (Figure 3). Activation in cells transfected with IR-B or IR-B-VFP was detectable by immunofluorescence and Western blot. By contrast, non-transfected cells (see cells without GFP signal in Figure 3C) or cells transfected with the empty vector [31] did not show detectable levels of activation. Activation of Mut-GFP was also analyzed after 5 or 15 min of rhIns stimulation and no activation was detected (Additional file 1: Figure S1).

**Figure 4:**
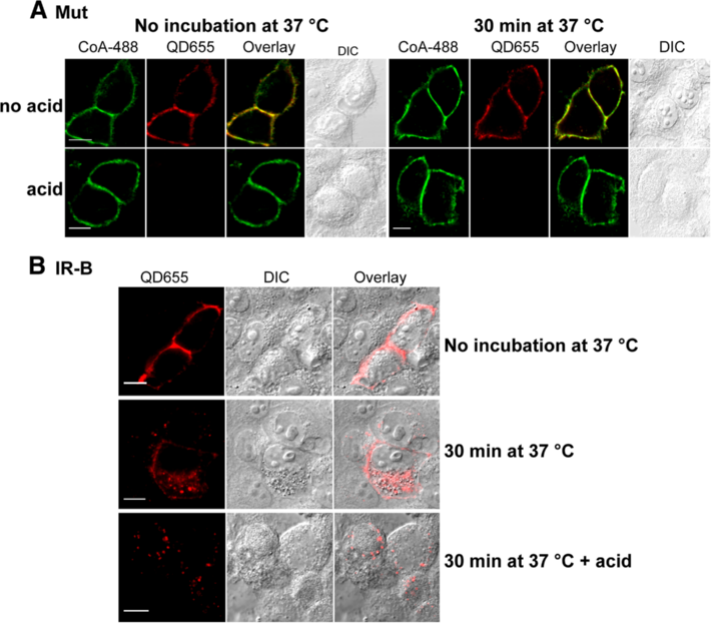
**Mutant retention at the plasma membrane. A**. HeLa cells expressing Mut labeled with 0.2 μM ACP-S and 1 μM CoA-488 were incubated with 50 nM BAC-Ins and 1 nM QD655. Cells were fixed or acid treated before fixation. Right panel: cells were incubated at 37°C for 30 min and fixed with or without acid treatment. **B**. Cells expressing IR-B were labeled with 50 nM BAC-Ins and 1 nM QD655, incubated or not at 37°C for 30 min and fixed or previously acid treated. Samples were imaged by confocal microscopy. Scale bars: 10 μm.

Insulin binding leads to the phosphorylation of IR triggering different signaling pathways. However, IR signaling is not limited to its activation at the membrane. Activated ligand-receptor complexes are internalized into endosomes where the IR kinase would be able to phosphorylate substrates that are spatially distinct from those accessible at the plasma membrane. Therefore, we studied the endocytosis of the tagged IR after insulin binding ACP-S acts optimally at 37°C and at this condition receptors could be recycled or internalized. We tested two different labeling temperatures (15°C and room temperature) finding that room temperature allowed both ACP and QD labeling with undetected internalization (Additional file 2: Figure S2A). Cells expressing Mut were labeled at room temperature with BAC-Ins and QD655, incubated at 37°C and directly fixed or acid treated to remove the ligand bound to the IR at the cell surface [31,32,35]. After acid treatment no QD655 signal was detected inside the cells expressing the mutant (Figure 4A) suggesting that endocytosis was blocked. In contrast, cells expressing wt IR-B showed normal endocytosis (Figure 4B) [30].

### Mut dimerizes with functional IR at the plasma membrane and blocks its internalization

**Figure 5:**
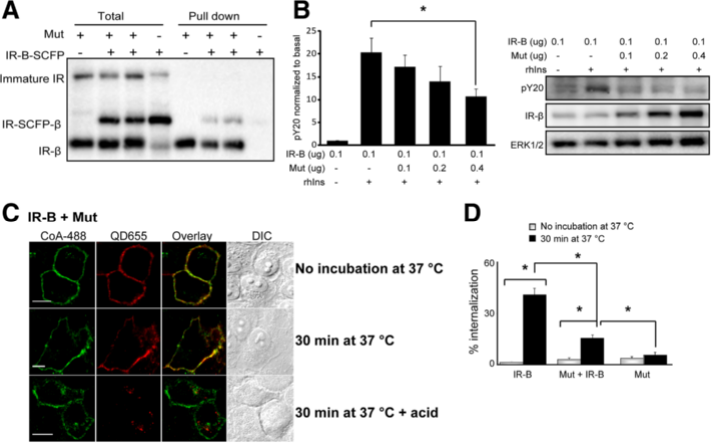
**Dimers wild type / mutant at the plasma membrane and signaling. A**. HeLa cells expressing Mut, IR-B-SCFP or both were treated with 2 μM ACP-S and 5 μM CoA-biotin for 30 min. Lysates were incubated with SA-agarose beads for 1 h at 4°C. Precipitates and total fractions were analyzed by Western blot with anti-IR-β subunit. **B**. HeLa cells were co-transfected with 0.1 μg pcDNA3-IR-B and different amounts of the mutant or EV, stimulated with 100 nM rhIns for 5 min and assayed by Western blot. Quantification was performed by densitometry measuring pY20 signal with respect to the first lane (basal) (*: *p*<0.05, *n*=4). **C**. HeLa cells co-expressing Mut and IR-B were labeled with 2 μM ACP-S and 2 μM CoA-488 and then incubated with 50 nM BAC-Ins and 1 nM QD655. Cells were directly fixed or incubated for 30 min at 37°C and then fixed or acid treated before fixation. Samples were imaged by confocal microscopy. Scale bars: 10 μm. **D**. Internalization analysis. Results are expressed as the mean ± s.e.m (*p*<0.005; *n*=6-32 cells).

We biotinylated the IR in cells co-expressing IR-B-SCFP and Mut and performed a SA-pull down assay followed by Western blot to confirm the presence of IR-B/Mut dimers at the plasma membrane. Transfected cells were incubated with 2 μM ACP-S and 1 μM CoA-biotin and correct surface modification was observed by labeling cells with 1 nM SA-550. The modification was specific and only present at the surface (Additional file 2: Figure S2B). The presence of IR-B-SCFP in the pull down fraction indicates that Mut was able to dimerize with wild type receptors (Figure 5A). Densitometric and statistical analysis showed that dimerization occurred stochastically without differences between mutant or wild type receptors (Additional file 3: Supplemental Experimental Procedures).

To analyze Mut's effect on insulin signaling we first evaluated IR phosphorylation in cells co-expressing IR-B and increasing amounts of Mut. Western blot experiments showed that IR phosphorylation was reduced by Mut in a concentration dependent manner (Figure 5B) suggesting a dominant negative effect.

Cells co-expressing Mut and wild type IR-B showed that Mut blocks insulin-IR complex endocytosis (Figure 5C). Cells with high levels of mutant expression (revealed by CoA-488 signal) showed a low proportion of internalized BAC-Ins-QD655 compared with cells with a low expression where a high endocytosis degree was observed. We quantified the QD655 signal inside the cell, at the membrane and the percentage of internalized QD (Additional file 4: Figure S3). The mutant's effect on internalization was analyzed in cells co-expressing IR-B with similar expression levels (CoA-488>1600 cts). While IR-B is internalized (49±5%; *n*=14), the mutant does not (7±2%; *n*=6) and retained IR-B at the membrane (18±2%; *n*=32) when they are co-expressed (Figure 5D). We further confirmed that no internalization took place at later time points (15, 45 and 150 min) (Additional file 5: Figure S4). By contrast IR-B and IR-B-VFP showed almost complete insulin internalization after 150 min [31,32].

The IR phosphorylation pattern regulates its internalization and is the proposed mechanism for the divergence of the mitogenic and metabolic signaling. It was postulated that its kinase activity modulation leads to the differential balance between metabolic and mitogenic response [36].

### Mut blocks insulin induced AP-1 activity without affecting Akt activation

**Figure 6:**
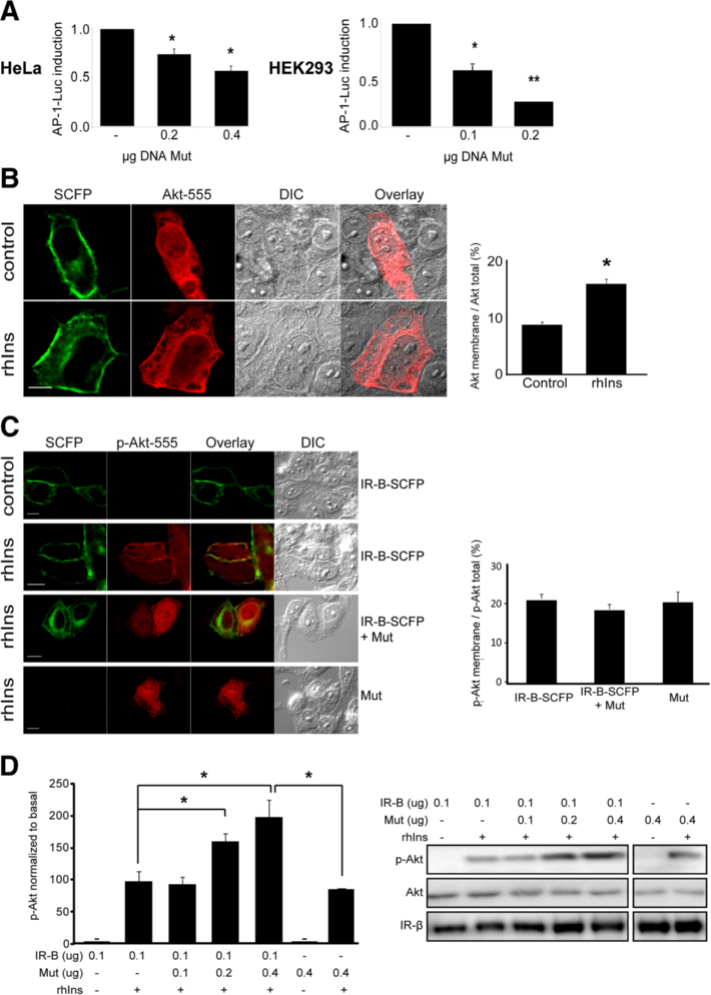
**Mutant IR blocks insulin induced AP-1 activity without affecting Akt activation. A**. HeLa or HEK293 cells co-expressing AP1-Luc, IR-B and different amounts of the mutant were starved for 24 h and then stimulated with 100 nM rhIns for 16 h. Luciferase activity was measured and fold induction was calculated (*: *p*≤0.03, **: *p*=0.003; *n*≥3). **B**. Akt translocation to the plasma membrane: HeLa cells co-expressing IR-B-SCFP and Akt-HA were starved overnight and then stimulated with 100 nM rhIns for 5 min. After fixation samples were stained with anti-Akt and a secondary antibody conjugated with Alexa fluor 555. Images were acquired by confocal microscopy and quantification of Akt re-distribution was evidenced (*: *p*<10^-6^; *n*=34-40 cells). Scale bars: 10 μm. **C**. Similar experiment was performed using anti-phospho-Akt in cells co-expressing Akt-HA with: *i)* IR-B-SCFP, *ii)* Mut *iii)* both (*n*=15-23 cells). **D**. Cells co-transfected with 0.1 μg pcDNA3-IR-B and different amounts of the mutant or EV were stimulated with 100 nM rhIns for 5 min and assayed by Western blot. Quantification was performed by densitometry measuring phospho-Akt signal normalized to the first lane (basal) (*: *p*<0.05, *n*≥3). ´p´ means phospho-antibodies. Results are expressed as the mean ± s.e.m.

To test the effect of the membrane retention downstream the IR, we measured AP-1 transcriptional activity induced by insulin using a luciferase reporter assay. Cells co-expressing AP-1-Luc, IR-B and increasing amounts of Mut were stimulated with 100 nM rhIns for 16 h. AP-1 induction was significantly decreased by Mut in a concentration dependent manner (Figure 6A). To further analyze this effect on endogenous IR, we measured AP-1 activity in response to insulin in HEK293 cells, which express predominantly IR-A. Increasing amounts of Mut significantly reduced insulin induction of AP-1 activity (0.1 μg DNA: 44% of reduction; 0.2 μg DNA: 92% of reduction) (Figure 6A). These results indicate that Mut-IR acts as a dominant negative in the pathway leading to AP-1 activation.

It is known that Akt translocates to the plasma membrane where interacts with the kinases that induce its activation to regulate glucose metabolism, differentiation, protein synthesis and cell survival and proliferation [37-39]. We confirmed Akt recruitment to the membrane after insulin activation by quantitative immunofluorescence. Cells co-expressing IR-B-SCFP and Akt-HA (Akt fused to the human influenza hemagglutinin tag, HA) were stimulated with rhIns and the proportion of Akt at the membrane was quantified. As expected, Akt translocated to the plasma membrane in response to insulin (control: 90±0.5%; rhIns: 160±0.9%; *n*=34-40 cells; *p*<10^-5^) (Figure 6B). Expression of the mutant alone or together with IR-B-SCFP did not change the intracellular re-distribution of Akt after insulin stimulation (Figure 6C). Moreover, Western blot experiments showed that expression of the mutant increased Akt activation in a concentration dependent manner (Figure 6D). This effect is not observed for ERK1/2 activation (Additional file 6: Figure S5).

There are at least seven tyrosines subjected to phosphorylation upon insulin binding [40]: Tyr965 and Tyr972 are phosphorylated in *cis* and involved in substrates selection [41]; Tyr1328 and Tyr1334 play a key role in mitogenic signaling and are not involved in metabolism and Akt signaling [42,43]; Tyr1158, Tyr1162 and Tyr1163 are the first residues to be phosphorylated in *trans*, known to mediate kinase activation and internalization [44]. It has been postulated that the degree of IR kinase activation leads to the differential balance between metabolic and mitogenic response [36]. IR internalization is required for Shc/MAPK pathway activation but not for IRS-1 and Akt phosphorylation suggesting that these molecules could be activated at the membrane [37-39,45-47]. We hypothesize that the mutant, acting both in *cis* or *trans*, could be affecting the phosphorylation pattern of the hertero- and homo-dimers blocking IR internalization and favoring membrane signaling.

## Conclusions

These results suggest that the mutant is acting as a selective dominant negative, blocking internalization and signaling from endosomes without affecting Akt activation at the cell surface. Our results are in agreement with the model proposing that the internalization dynamics is crucial for specific IR signaling suggested from different independent studies and reviewed by Jensen and De Meyts [47]. The mutant binds insulin but fails to get activated. When this chimera dimerizes with wild type IR, hybrid receptors fail to get fully phosphorylated and are consequently retained at the plasma membrane, promoting Akt activation and inhibiting endosomal signaling.

## Methods

### Materials

rhIns was provided by Laboratorios Beta (Argentina). BAC-Ins was from Sigma (Germany). Mouse monoclonal anti-IR-β subunit, rabbit monoclonal anti-phospho-Akt (Ser473), anti-Akt, anti-ERK1/2 and anti-phospho-IR-β subunit (Tyr1361) were from Cell Signaling Technology (Beverly, MA). Mouse monoclonal anti- pY20 was from BD Transduction Laboratories (San Jose, CA). Mouse monoclonal anti-PY99 was from Santa Cruz Biotechnology (Santa Cruz, CA). QD655 and secondary antibodies conjugated with Alexa fluor 555 were from Molecular Probes, Invitrogen (Eugene, OR). Lipofectamine Reagent 2000 was from Invitrogen (Carlsbad, CA). Buffers and enzymes for cloning were from New England Biolabs (Ipswich, UK), Promega (Madison, WI) and Invitrogen (Carlsbad, CA). Ni-NTA resin was from QIAGEN (Valencia, CA). The plasmid containing luciferase reporter gene downstream of 7 binding sites for AP-1 (p-AP-1-Luc) and Akt-HA were generously provided by Dr Omar Coso and Dr Anabella Srebrow (IFIBYNE, Argentina) respectively. The plasmids pcDNA3-IR-B, pcDNA3-IR-B-GFP and pcDNA3-IR-B-SCFP were recently described [31,32].

### Generation of expression constructs

*pcDNA3-IR-B-A1×3-GFP (Mut-GFP):* oligonucleotides with the A1 sequence (A1BamHI5’: 5´-gatcttggtgactctctggacatgctggagtggagtctgatgggt-3´; A1BamHI3’: 5´-gatcacccatcagactccactccagcatgtccagagagtcaccaa-3´) were annealed for 5 min at 100°C cooling it to RT (500 mM NaCl, 10 mM EDTA, 100 mM Tris–HCl pH 7.5). Presence of double strand DNA with sticky ends for *BamH*I was tested by an absorption spectrum. Annealing product was treated with T4 PNK. The pcDNA3-IR-B-GFP was digested with *BamH*I restriction enzyme and ligated with the A1 tag with the *BamH*I ends. *pcDNA3-IR-B-A1×3 (Mut):* Mut-GFP was digested with *Nhe*I and *Apa*I restriction enzymes, treated with Klenow and T4 DNA polymerase and then re-ligated to generate Mut.

### Cell culture and transfections

HeLa cells were maintained in DMEN (GIBCO; Grand Island, NE) supplemented with 100 U/ml penicillin, 100 μg/ml streptomycin and 10% fetal bovine serum (FBS) at 37°C in 5% CO_2._ HEK293 cells were similarly maintained adding 1 mM sodium pyruvate. Cells were plated at 1×10^5^ cells/well density in 24 well plates (luciferase assays) or onto 12 mm glass coverslips (microscopy) or at 2.5×10^5^ cells/well density in 12 wells plates (Western blot) one day before transfection in DMEM/10% FBS. Cells were transfected with Lipofectamine Reagent 2000 according to manufacturer protocols.

### Western blots

Following stimulation with 100 nM rhIns cells were lysed in 100 mM Tris–HCl pH 6.8, 4% SDS, 0.2% Bromophenol blue, 20% glycerol, 200 mM β-mercaptoethanol, vortexed for 20 sec and boiled for 5 min. After 10% SDS-PAGE and transfer, PDVF membranes were blocked in 5% non-fat dried milk in 0.1% Tween-TBS buffer (TTBS) for 1 h, washed and incubated overnight at 4°C with primary antibodies diluted in 5% BSA/TTBS. The following day, membranes were incubated with secondary antibodies for 1 h and developed by chemiluminescence. Quantification was performed by densitometry using ImageJ plugins (NIH).

### Luciferase reporter assay

Cells seeded onto 24 well plates the day before were transfected using 0.3 μg pcDNA3-IR-B (HeLa cells) or EV (HEK293 cells), 0.05 μg pAP-1-Luc and different amounts of Mut. After 24 h, cells were starved one day, and then stimulated for 16 h with 100 nM rhIns. Luciferase activity was determined using Luciferase Reactive and Reporter Lysis Buffer from Promega (Madison, WI) and normalization to the control (non-stimulated cells) was performed.

### Expression and purification of ACP-S

#### ***Protein expression***

TOP 10 *Ecoli* cells (araBADC- and araEFGH+) were electroporated with pBAD-ACPwt-S plasmid (provided by T.M. Jovin and D.J. Arndt-Jovin, Max Planck Institute for Biophysical Chemistry, Germany), plated on Luria Broth agar and incubated at 37°C overnight. The next day, a 2×YT starting culture was inoculated overnight at 37°C. We diluted (1/200) the starting culture and growth it at 37°C to an optical density of 0.8. The expression was induced with 0.1% (w/v) L-(+)-arabinose (SIGMA-Aldrich, Germany)and incubation at 30°C overnight and the next day cells were harvested. The pellet was resuspended (10 ml/g pellet) in Lysis Buffer (80 mM imidazole pH 8.0, 250 mM NaCl and protease inhibitor) and was sonicated 3×15 sec. Then 1% Triton X-100 was added and the suspension was stirred for 15 min at 4°C. Lysates were centrifuged at 9,500 r.p.m. for 30 min at 4°C collecting the supernatant.

#### ***Protein purification by Ni-NTA chromatography***

The cleared lysate was incubated with Ni-NTA resin (20 ml resin per 500 ml lysate) at 4°C for 1 h with stirring. The resin was then loaded in a 10 ml syringe, washed with Wash Buffer 1 (500 mM NaCl, 100 mM imidazole pH 8.0) and then with Wash Buffer 2 (100 mM NaCl, 100 mM imidazole pH 8.0, 20% glycerol). Elution was performed with 100mM NaCl, 400 mM imidazole pH 8.0, 20% glycerol. Fractions were analyzed by SDS-PAGE and by measuring absorption at 280 nm. Fractions containing the protein were combined and stored at -80°C with 1 mM DTT after shock-freezing in liquid nitrogen.

### CoA derivatives synthesis

#### ***CoA-biotin***

CoA trilithium salt (FLUKA, St Louis, MO) (1 equivalent) was dissolved in 50 mM Tris–HCl pH 7.5 by adding 10 volumes of dimethyl sulfoxide (DMSO). This solution was combined with Biotin-Peo_2_-maleimide (Pierce, Rockford, IL) (1 equivalent) dissolved in DMSO at room temperature for 4 h. Product reaction was purified by Fast protein liquid chromatography (ÄKTA Purifier 100, GE Healthcare Life Science) with a reverse phase column C18 Kromasil MZ refill 100 (150 mm × 4 mm). Elution was performed with a gradient acetonitrile: water/0.1% trifluoroacetic acid from 0:100 to 60:40 in 45 min. The purified product was characterized by Matrix-Assisted Laser Desorption/Ionization using a time of flight ion detector (CEQUIBIEM, FCEN-UBA, Argentina) with matrix 3-hidroxipicolinic acid. Quantification was performed by spectrophotometry (ϵ_adenine260nm_=15,300 M^-1^cm^-1^). The product was lyophilised, resuspended in DMSO and stored at -20°C.

#### ***Fluorescent CoA***

ATTO-maleimide (488, 532, 550) (ATTO Tec GmbH, Germany) (2 equivalents) was dissolved in DMSO and CoA trilithium salt (1 equivalent) was dissolved in MES (2-(N-morpholino) ethanesulfonic acid) buffer pH 7.0 and one volume of DMSO. We combined both solutions at 20°C overnight. The product was purified by HPLC with a reverse phase column C18 Kromasil MZ semiprep 100 (250 mm × 8 mm) and the following elution gradient: *(i)* 2 min ammonium acetate: acetonitrile (97:3); *(ii)* 20 min from 97:3 to 40:60; *(iii)* 5 min from 40:60 to 0:100. The product identity was confirmed by mass spectrometry, spectrophotometry and by labeling cells expressing A1 tag with ACP-S. Fluorescent CoA was lyophilized, resuspended in DMSO and stored at -20°C.

### Label *in vivo* with ACP-S and CoA derivatives

Transfected cells were washed with Tyrode´s buffer (135 mM NaCl, 10 mM KCl, 10 mM MgCl_2_, 1mM CaCl_2_, 10 mM HEPES pH 7.2, 0.1% BSA) and incubated with 0.2 or 2.0 μM ACP-S and 1 μM CoA derivatives for 30 min at room temperature and finally washed four times with Tyrode´s CoA-biotin modification was followed by a labeling step with 1 nM SA-atto550 for 15 min at room temperature. Then cells were fixed in cold methanol for 30 min at -20°C and mounted for imaging.

### Labeling *in vivo* with QD655 and BAC-Ins and internalization

Before the experiment cells were starved overnight and then incubated with 50 nM BAC-Ins for 15 min at room temperature, washed and incubated with 1 nM QD655 for 10 min, washed and either fixed in 37% paraformaldehyde (PFA) on ice for 20 min or incubated at 37°C in DMEM for different periods before fixation. When acid treatment was applied, cells were incubated for 5 min at room temperature with acid solution (0.1 M Na-glycine pH 3.0, 0.5 M NaCl) [28-30] and then fixed Imaging was performed in PBS.

### Immunofluorescence

After overnight starvation, transfected cells were stimulated with 100 nM rhIns for 5, 10 or 15 min, washed with cold PBS and immediately fixed in cold methanol for 30 min at -20°C, blocked with PBS/0.3% Triton X-100/1% BSA for 1 h at 37°C and incubated with anti-phospho-IR-β subunit (0.3 μg/ml), anti-PY99 (1 μg/ml), anti-Akt (1/400) or anti-phospho-Akt (1/200) overnight at 4°C. The following day samples were incubated with a secondary antibody conjugated with Alexa fluor 555 or Cy3 for 1 h at 37°C, washed and mounted for imaging.

### Microscopy

Confocal microscopes were Olympus Fluoview FV1000 with a UPLSAPO 60×1.2 N.A. water immersion objective or Zeiss LSM510 Meta with a C-Apochromat 63×1.2 N.A. water immersion objective or a Plan-Apochromat 63×1.4 N.A. oil immersion objective. Excitation and emission filters were as follows:

*SCFP and Alexa fluor 555*: Excitation: SCFP, 405 nm; Alexa fluor 555, 543 nm. Emission: SCFP, band-pass (BP): 430–470 nm; Alexa fluor 555, BP: 560–620 nm.

*CoA-488 and QD655*: Excitation: CoA-488, 488 nm; QD655, 405 nm. Emission: CoA-488, BP: 505–525 nm; QD655, BP: 640–670 nm.

*GFP and Alexa fluor 555*: Excitation: GFP, 488 nm; Alexa fluor 555, 543 nm. Emission: GFP, BP: 505–525 nm; Alexa fluor 555, BP: 560–620 nm.

*CoA-488 and Alexa fluor 555*: Excitation: CoA-488, 488 nm; Alexa fluor 555, 543 nm. Emission: CoA-488, BP: 505–525 nm; Alexa fluor 555, BP: 560–620 nm.

*GFP and CoA-532:* Excitation: GFP, 488 nm; CoA-532, 532 nm. Emission: GFP, BP: 500/20 nm; CoA-532, 563–606 nm.

*GFP, CoA-532 and QD655:* Excitation: GFP, 488 nm; CoA-532, 532 nm; QD655, 488 nm. Emission: GFP, BP: 500/20 nm; CoA-532, BP: 565–615 nm; QD655, long-pass: 650 nm.

Wide field microscope was Olympus IX71 with a 40× 1.15 N.A. water immersion objective, a mercury arc lamp excitation, suitable filters and a camera Hamamatsu Orca CCD C4742-95.

### Image processing

Confocal images were processed with Matlab and ImageJ. Background was subtracted and in some cases a median filter was applied only for presentation.

### Internalization analysis

#### ***Segmentation (membrane-interior)***

Channel background (median) was subtracted. Cell segmentation was performed manually and *pre-membrane* was defined as the difference image of the cell and binary erosion (iterations: 5–20; alternating connectivity). The *pre-interior* was defined as the difference between *cell* and *pre-membrane.* With a *mask* marking red pixels (QD655) *membrane* was defined as the product *mask* × *pre-membrane*, and *interior* as the product *mask* × *pre-interior.*

#### ***Endocytosis estimation***

Values in *membrane* and *interior* were summed for both channel, also sizes were measured In order to compute the relative amount of internalized red fluorescence we estimated QD_*total*_ (QD_*membrane*_+QD_*interior*_) and calculated the ratio QD_*iinterior*_/QD_total_ for each cell. Expression levels were estimated as the mean of the CoA-488 signal (sum of CoA-488/cell size). Cells with similar CoA-488 level were considered.

### Quantification of Akt distribution

Each cell was segmentated similarly using SCFP signal, red signal was measured in each region and Akt_membrane_/Akt_total_ ratio was calculated.

### Pull down experiments

Cells expressing the mutant alone or in combination with IR-B-SCFP were growth on six well plates. Cells were incubated with 2 μM ACP-S and 5 μM CoA-biotin for 30 min at room temperature and after 4 washes with PBS were lysed (50 mM Tris–HCl pH 7.5, 1 mM EDTA, 1 mM EGTA, 150 mM NaCl, 1% NP40, 1 mM MgCl_2_, 0.1% SDS, proteases inhibitors). Lysates were incubated with SA-agarose beads for 1 h at 4°C and centrifuged for 1 min. Supernatants were discarded and beads were washed with the same buffer twice. Beads were resuspended in loading buffer (100 mM Tris–HCl pH 6.8, 4% SDS, 0.2% bromophenol blue, 20% glycerol, 200 mM β-mercaptoethanol, 1mM DTT) as well as aliquots of total fractions (4% total lysate volume). Samples were boiled for 5 min and analyzed by Western blot.

### Statistics

Results were expressed as the mean ± s.e.m. *p* values were estimated using Student´s T test (2 tails).

For supplementary methods see Additional file 3.

## Abbreviations

ACP: Acyl carrier protein; ACP-S: Acyl carrier protein syntasa; BAC-Ins: Biotin amido caproyl insulin; BP: Band-pass; BSA: Bovine serum albumin; CoA: Coenzyme A; DMSO: Dimethyl sulfoxide; EV: Empty vector; FBS: Fetal bovine serum; FnIII-1: First Fibronectin type III domain; GFP: Enhanced green fluorescent protein; IGF: Insulin like growth factor; IGF-IR: Insulin like growth factor I receptor; IR: Insulin receptor; IR-A: Insulin receptor isoform A; IR-B: Insulin receptor isoform B; M: Manders coefficient; N.A.: Numerical aperture; PFA: Paraformaldehyde; QD: Quantum dot; rhIns: Recombinant human insulin; SCFP: Super cyan fluorescent protein; s.e.m.: Standard error from the mean; SYFP: Super yellow fluorescent protein; VFP: Visible fluorescent protein.

## Competing interests

The authors declare that they have no competing interests.

## Authors’ contributions

JG conceived the project, designed and performed all the experiments, analyzed the data and wrote the manuscript. EAJE conceived the project, designed the experiments and analyzed the data. FCL conceived the project, designed the experiments, analyzed the data, and wrote the manuscript. All authors read and approved the final manuscript with the exception of the deceased, Elizabeth A. Jares Erijman.

## Supplementary Material

Additional file 1: Figure S1 Mut-GFP is not activated after 15 min of stimulation. HeLa cells were transfected with Mut-GFP (A) or IR-B-GFP (B) and after overnight starvation they were stimulated with 100 nM rhIns for 5 or 15 min and fixed. Immunofluorescence assays were performed with anti-phospho-tyrosine (P99) and a secondary antibody conjugated with Cy3. Scale bars: 10 μm. Click here for file

Additional file 2: Figure S2 Effect of the temperature in the internalization during labeling and CoA-biotin labeling. A. HeLa cells expressing IR-B were labeled with BAC-Ins and QD655 at room temperature or at 15°C, fixed and imaged by confocal microscopy. Images were quantified as described in experimental section and the percentage of internalization was calculated. Results are expressed as the mean ± s.e.m. B. HEK293 cells expressing Mut-GFP were incubated with 1 μM CoA-biotin with or without 2 μM ACP-S and then labeled with 1 nM SA-550. Fixed cells were imaged by wide field microscopy. Scale bars: 5 μm. Click here for file

Additional file 3 Supplemental Experimental Procedures. Click here for file

Additional file 4: Figure S3 Effect of expression level on the internalization. Quantification of BAC-Ins-QD655 internalization was performed in cells co-expressing Mut and IR-B after 30 min at 37°C depending on the fluorescence levels of CoA-488. Cells were classified in *high labeling* (CoA-488>1600 cts) and *low labeling* (CoA-488<900 cts). Results are expressed as the mean ± s.e.m. (*: *p*=0.01; *n*=8 cells). Click here for file

Additional file 5: Figure S4 Mut-GFP endocytosis over time. HeLa cells expressing Mut-GFP labeled with 0.2 μM ACP-S and 1 μM CoA-532 were incubated with 50 nM BAC-Ins and 1 nM QD655. Cells were incubated at 37°C for 15, 45 or 150 min and then fixed. Samples were imaged by confocal microscopy. Scale bars: 10 μm. Click here for file

Additional file 6: Figure S5 Mut effect on ERK1/2 activation. HeLa cells co-transfected with 0.1 μg pcDNA3-IR-B and different amounts of the mutant or EV were stimulated with 100 nM rhIns for 5 min and assayed by Western blot. Quantification was performed by densitometry measuring phospho-ERK1/2 signal normalized to the basal (*: *p*<0.05, *n*≥3). ´p´ means phospho-antibodies. Results are expressed as the mean ± s.e.m. Click here for file
