# Supplementary figures and images for: Insulin receptor membrane retention by a traceable chimeric mutant
Source: Cell Commun Signal. 2013 Jun 27;11:45. doi: 10.1186/1478-811X-11-45 (PMC3707766; doi:10.1186/1478-811X-11-45)

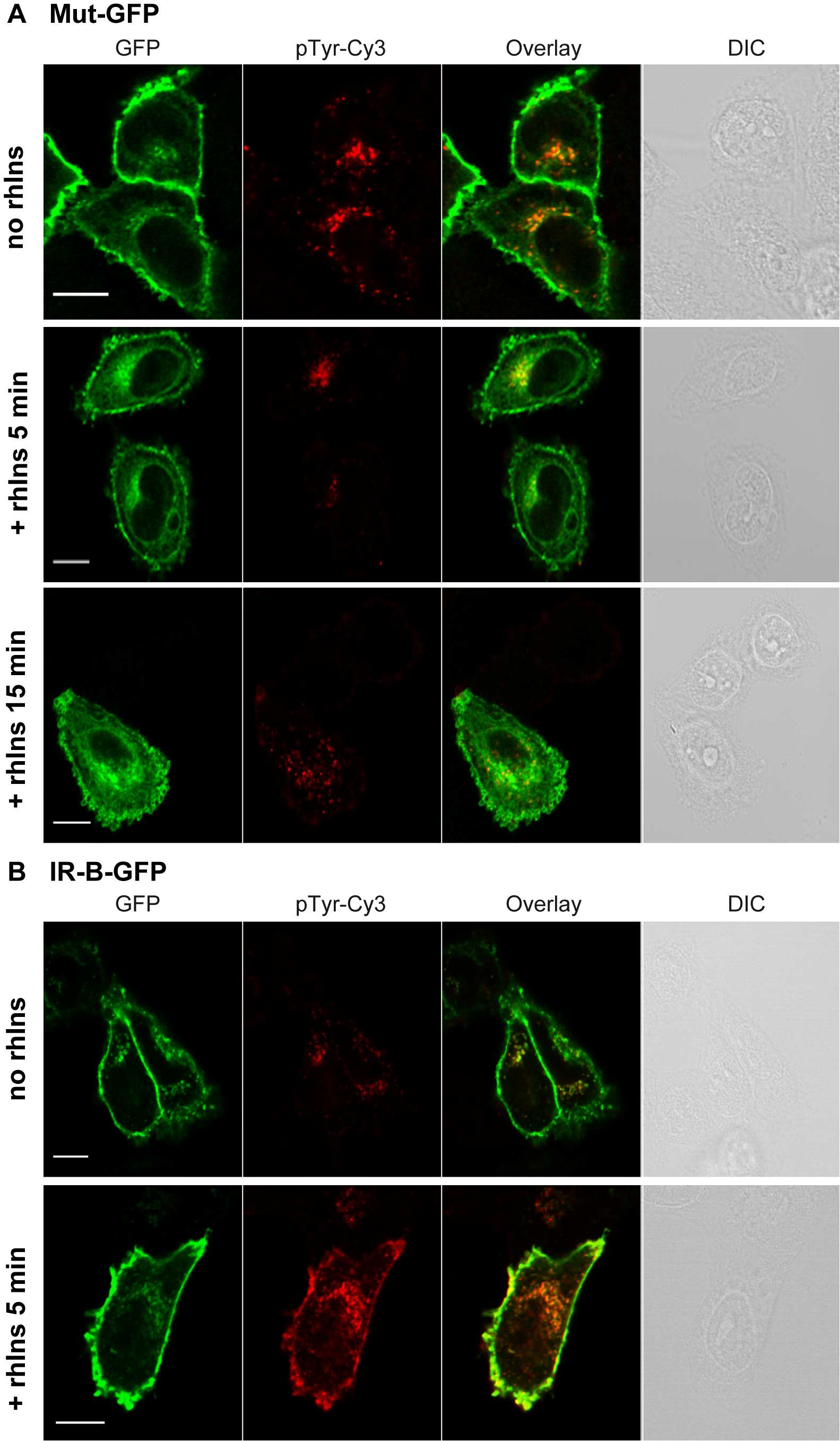

Supplement: Additional file 1: Figure S1 — Mut-GFP is not activated after 15 min of stimulation. HeLa cells were transfected with Mut-GFP (A) or IR-B-GFP (B) and after overnight starvation they were stimulated with 100 nM rhIns for 5 or 15 min and fixed. Immunofluorescence assays were performed with anti-phospho-tyrosine (P99) and a secondary antibody conjugated with Cy3. Scale bars: 10 μm. [file 1478-811X-11-45-S1.tiff]

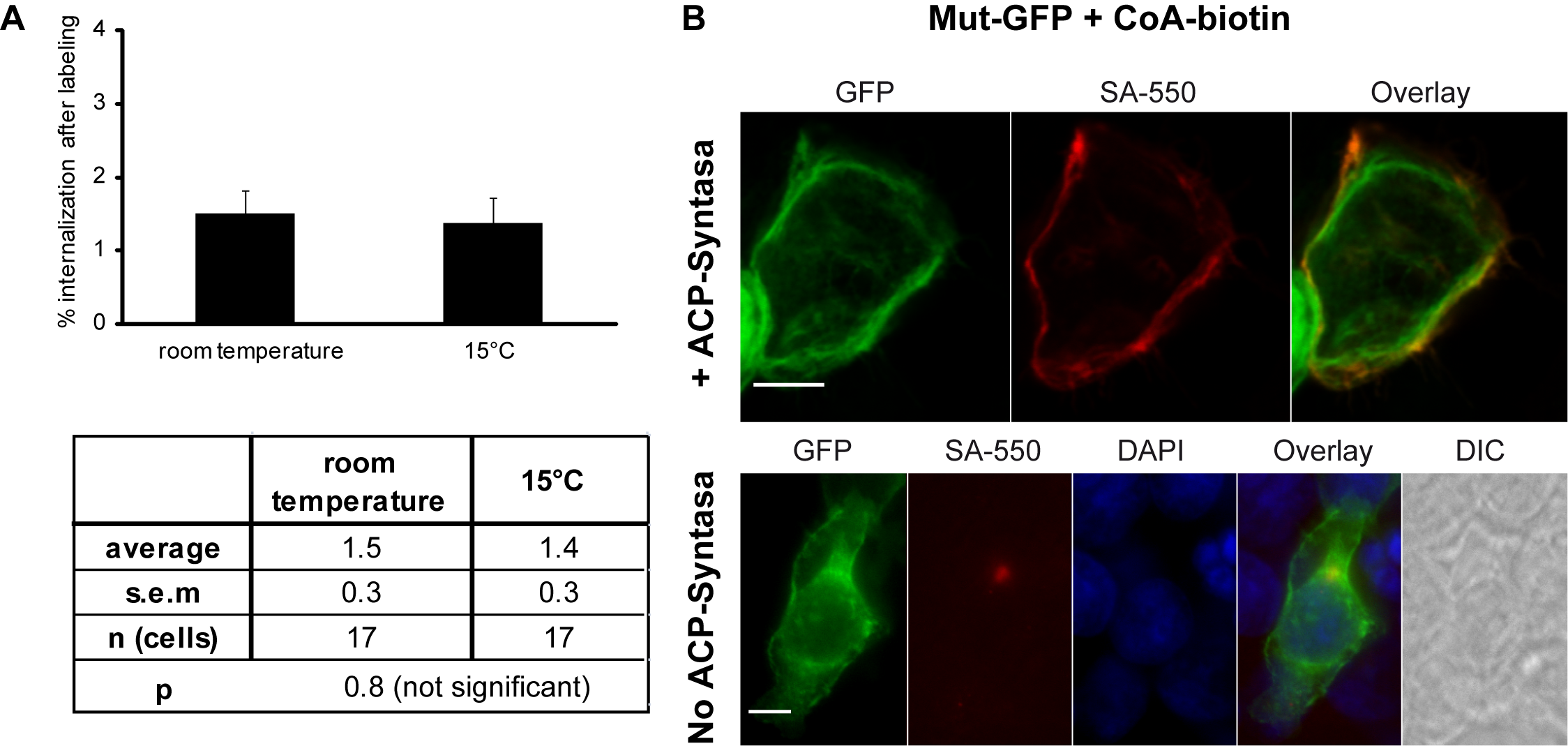

Supplement: Additional file 2: Figure S2 — Effect of the temperature in the internalization during labeling and CoA-biotin labeling. A. HeLa cells expressing IR-B were labeled with BAC-Ins and QD655 at room temperature or at 15°C, fixed and imaged by confocal microscopy. Images were quantified as described in experimental section and the percentage of internalization was calculated. Results are expressed as the mean ± s.e.m. B. HEK293 cells expressing Mut-GFP were incubated with 1 μM CoA-biotin with or without 2 μM ACP-S and then labeled with 1 nM SA-550. Fixed cells were imaged by wide field microscopy. Scale bars: 5 μm. [file 1478-811X-11-45-S2.tiff]

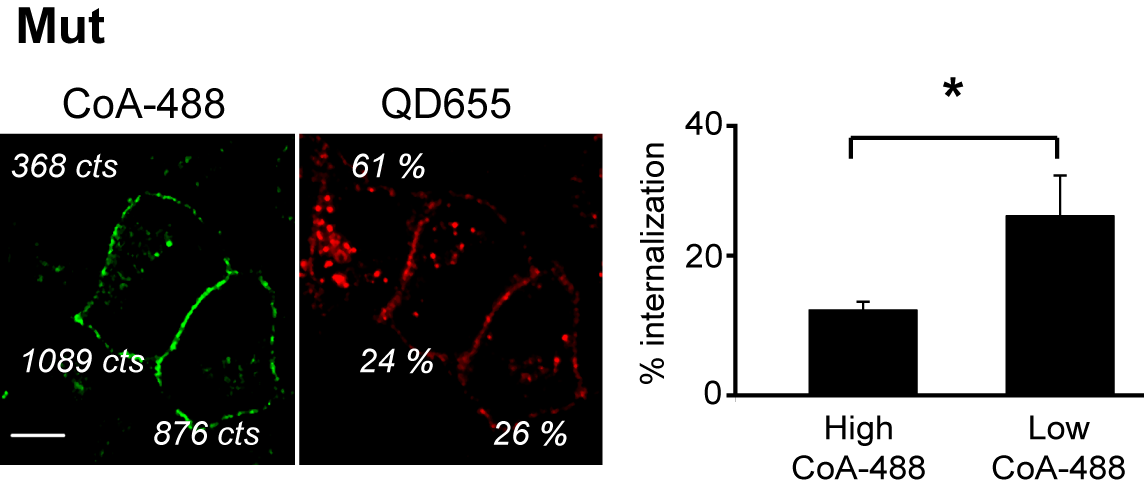

Supplement: Additional file 4: Figure S3 — Effect of expression level on the internalization. Quantification of BAC-Ins-QD655 internalization was performed in cells co-expressing Mut and IR-B after 30 min at 37°C depending on the fluorescence levels of CoA-488. Cells were classified in high labeling (CoA-488>1600 cts) and low labeling (CoA-488<900 cts). Results are expressed as the mean ± s.e.m. (*: p=0.01; n=8 cells). [file 1478-811X-11-45-S4.tiff]

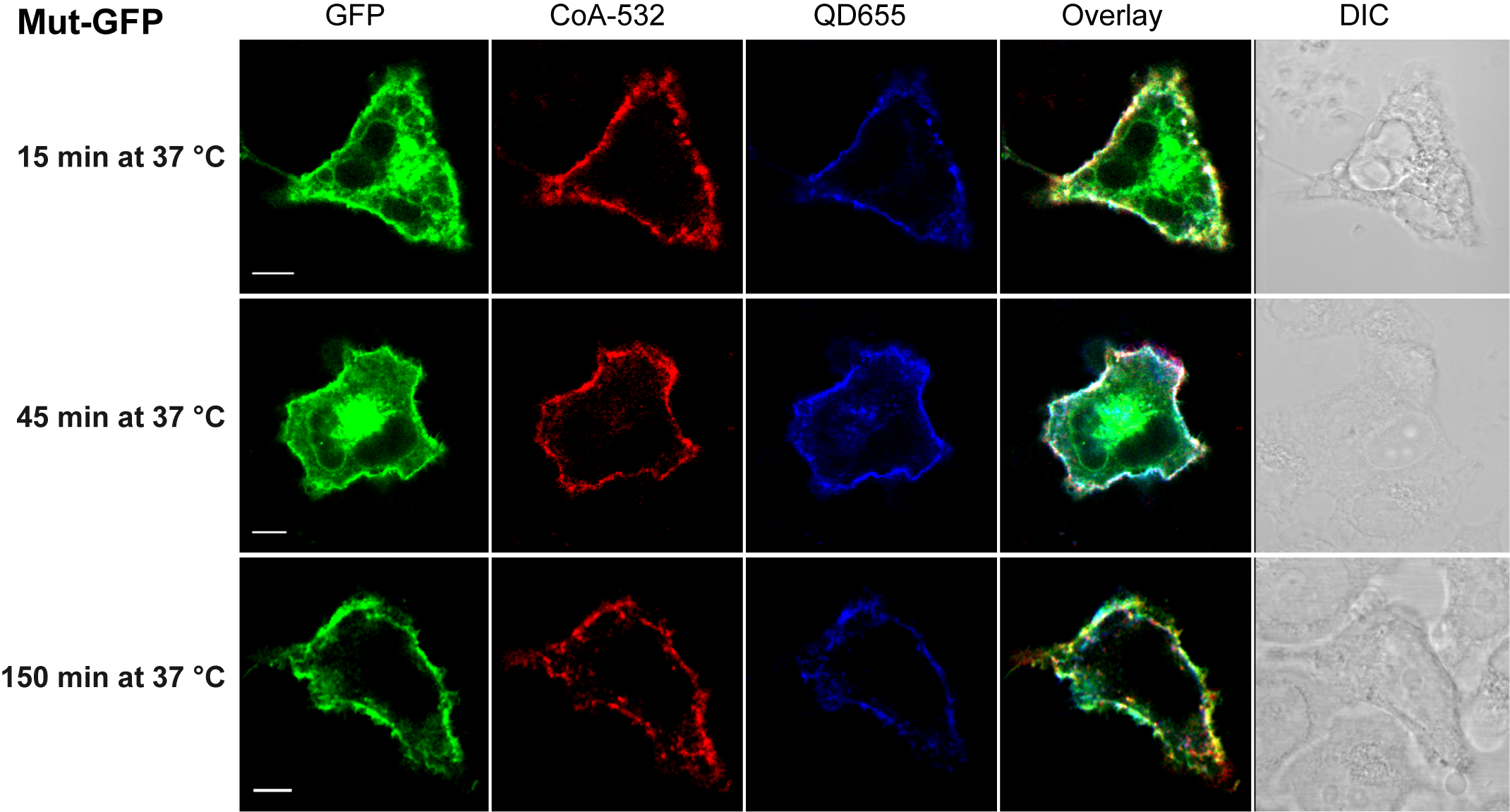

Supplement: Additional file 5: Figure S4 — Mut-GFP endocytosis over time. HeLa cells expressing Mut-GFP labeled with 0.2 μM ACP-S and 1 μM CoA-532 were incubated with 50 nM BAC-Ins and 1 nM QD655. Cells were incubated at 37°C for 15, 45 or 150 min and then fixed. Samples were imaged by confocal microscopy. Scale bars: 10 μm. [file 1478-811X-11-45-S5.tiff]

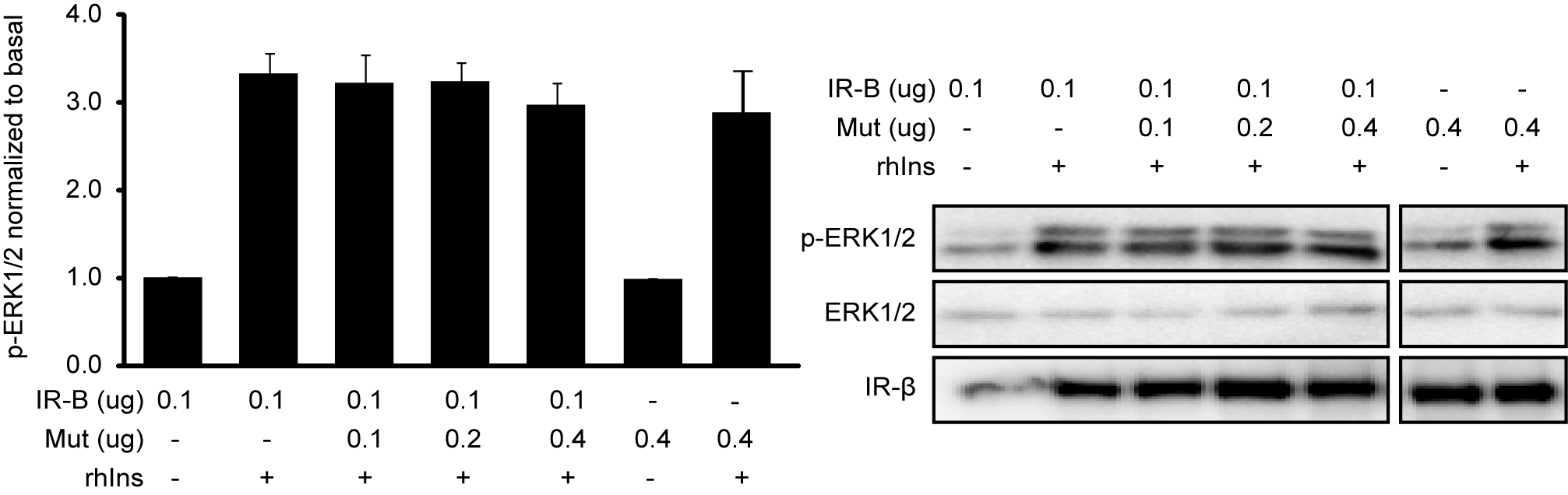

Supplement: Additional file 6: Figure S5 — Mut effect on ERK1/2 activation. HeLa cells co-transfected with 0.1 μg pcDNA3-IR-B and different amounts of the mutant or EV were stimulated with 100 nM rhIns for 5 min and assayed by Western blot. Quantification was performed by densitometry measuring phospho-ERK1/2 signal normalized to the basal (*: p<0.05, n≥3). ´p´ means phospho-antibodies. Results are expressed as the mean ± s.e.m. [file 1478-811X-11-45-S6.tiff]
