# Supplementary material for: Insulin receptor membrane retention by a traceable chimeric mutant
Source: Cell Commun Signal. 2013 Jun 27;11:45. doi: 10.1186/1478-811X-11-45 (PMC3707766; doi:10.1186/1478-811X-11-45)
Supplement: Additional file 3 — Supplemental Experimental Procedures. [file 1478-811X-11-45-S3.doc]

**SUPPLEMENTAL EXPERIMENTAL PROCEDURES**

We evaluated the probability of hetero-dimerization between Mut and IR-B-SCFP as compare with homo-dimer formation. We measured the densitometry ratio between the bands of Mut and IR-B-SCFP in the pull down fraction and we normalized it to the ratio in the total fractions. Stoichiometric dimer formation assuming equal expression would form: 2 IR-B-SCFP/Mut, IR-B-SCFP/IR-B-SCFP and Mut/Mut. Since expression levels are variable we normalized IR-BSCF/Mut ratio in the pull down fraction (r_exp_) to the expected ratio (r_heo_) considering expression levels in the total fractions (R).

R is the relative abundance of Mut (a) and IR-B-SCFP (b) (equation 1) calculated by densitometry from total fractions.

$\text{R=}\frac{\text{a}}{\text{b}}$ **[equation 1]**

The probabilities of hetero-dimer formation IR-B-SCFP/Mut (P_1_) or homo-dimer formation Mut/Mut (P_2_) are:

**[equation 2]**

**[equation 3]**

For each molecule of hetero-dimer IR-B-SCFP/Mut one molecule of IR-B-SCFP and one of Mut would be generated after breaking the disulfide bonds. For each molecule of homo-dimer Mut/Mut, two molecules of Mut and none of IR-B-SCFP would be obtained. Therefore, the expected abundance of each band in the pull down fraction can be written in terms of R by equations 4 and 5.

**[equation 4]**

**[equation 5]**

In consequence if the dimer formation follows the stoichiometry, the expected band ratio in the pull down fractions would be given by equation 6.

**[equation 6]**

We estimated r_theo_ and r_exp_ for five independent experiments and r_exp_/r_theo_=1.3 ±0.1 indicating that dimerization occurs stochastically without differences between mutant or wild type receptors
